# SARS-CoV-2 diagnostic testing in Africa: needs and challenges

**DOI:** 10.11604/pamj.2020.35.4.22703

**Published:** 2020-04-14

**Authors:** Yusuff Adebayo Adebisi, Gabriel Ilerioluwa Oke, Peter Sunday Ademola, Iwendi Godsgift Chinemelum, Isaac Olushola Ogunkola, Don Eliseo Lucero-Prisno III

**Affiliations:** 1 Faculty of Pharmacy, University of Ibadan, Ibadan, Nigeria; 2 Department of Medical Laboratory Science, Ladoke Akintola University of Technology, Oyo State, Nigeria; 3 Faculty of Pharmaceutical Sciences, University of Port Harcourt, Rivers State, Nigeria; 4 Department of Public Health, University of Calabar, Cross River State, Nigeria; 5 Department of Global Health and Development, London School of Hygiene and Tropical Medicine, United Kingdom

**Keywords:** 2019-nCoV, SARS-CoV-2, COVID-19, coronavirus, Africa, diagnostic testing, pandemic, global health

## To the editors of Pan African Medical Journal

Novel Coronavirus (also called COVID-19, or 2019-nCoV, or SARS-CoV-2) is an unprecedented pandemic [1]. As depicted by the upsurge of the number of SARS-CoV-2 cases and the rapid geographical expansion of the virus, it is evident that it is truly a global health threat and Africa is not an exception to this threat. As of March 31 2020, 5, 287 SARS-CoV-2 cases and 172 deaths have been reported in 48 African nations [2]. However, underreporting of numbers of cases may be due to diagnostic insufficiency, low testing capacity and most of all the fragile healthcare system on the continent. This letter emphasizes the needs and challenges of SARS-CoV-2 diagnostic testing in Africa. Globally, SARS-CoV-2 diagnostic testing is a challenge. However, the low testing in Africa is a further challenge as the healthcare capacity and human resources for health are limited to respond adequately to high caseload. The early-stage asymptomatic characteristic of the virus has enabled silent transmission which is a further concern to identify and test those infected. In lieu of this, there is a need for targeted large-scale testing and this can only be achieved through a more rapid, accurate and affordable diagnostic testing approach and scaling up laboratory testing capacity. However, laboratory testing is not without challenges as African nations have limited well-equipped laboratories that can cater for its population [3]. The dearth of clinical laboratory scientists on the continent [3] is also another challenge that is likely to contribute to the diagnostic insufficiency. This further reinforces the need for rapid diagnostic testing and the need to develop laboratory capacity and its human resources in Africa.

As of April 6 2020, South Africa has the highest number of cases in Africa. This can be supported by the proactiveness of the country in leading mass testing strategy. To date, it has tested more than 47,000 people [4]. On April 2 2020, Zimbabwe had tested only 316 people for the virus [5] while as of April 1 2020, Namibia, whose testing is done locally and in South Africa, has conducted only 306 tests [6]. Rwanda [7] and Kenya [8] are also struggling to meet up with its testing needs to fully understand the extent of the outbreak. Many African countries are still struggling to get more people tested. However, African nations can learn from South Africa by leveraging both the private and public sectors and fostering public-private partnerships to increase testing capacity. Scaling up laboratories testing capacity and widespread use of rapid and accurate diagnostic tools will not only reduce the spread of the virus but also ensure early detection of cases and early medical intervention. This will also contribute to a reduction in the case fatality ratio. With targeted large-scale testing, the progression of the pandemic, regional variation and how the virus affects people of different ages and genders can be measured and better understood. Even though WHO and Africa CDC are supporting countries in scaling up their laboratory capacity, it would be essential to complement efforts with rapid diagnostic tools that are accurate and affordable. As of April 4 2020, there are no approved validated rapid diagnostic testing devices that is approved for use across the continent. Upon availability, access should be prioritized by African governments.

Majority of the African nations rely on molecular testing (e.g quantitative RT-PCR testing), which is relatively slower compared to a typical rapid diagnostic tool and also face a challenge of limited laboratories and human resource for health. According to WHO interim guidance, emphasis was laid that not having laboratory-confirmed cases does not imply that a country is free from SARS-CoV-2 and can be a sign of insufficient testing and surveillance [9]. This insufficiency can also result in underreporting of cases. This happens to be the situation in many African countries. Laboratories across the continent still face the challenge of limited test kits resulting into formulation of guidelines to test only people that show symptoms, that recently visit high-risk countries or have exposure to an already infected person. This is worrisome in that the SARS-CoV-2 remains infectious among infected individuals without symptoms.

## Conclusion

Large-scale testing is not without challenges; however, the needs cannot be overemphasized. African context is unique and so must be its responses to the SARS-CoV-2 pandemic. Making testing more available and affordable, advocating for physical distancing, early isolation and mandatory quarantine, proper respiratory and hand hygiene and other precautionary measures while working on expanding the healthcare capacity will contribute to effective responses to the pandemic.

## Competing interests

The authors declare no competing interests.
